# Positive impact of social relationships fostered by physical and/or cognitive group activity on older people’s quality of life: PRISMA systematic review

**DOI:** 10.3389/fpsyg.2023.1166072

**Published:** 2023-09-12

**Authors:** Tiphanie Gonnord, David Clarys, Geoffroy Boucard, Catherine Esnard

**Affiliations:** ^1^Centre de Recherches sur la Cognition et l’Apprentissage, Département de Psychologie, Université de Poitiers, Université François Rabelais de Tours, CNRS, Poitiers, France; ^2^Laboratoire Mobilité Vieillissement Exercice (EA6314), Faculté des Sciences du Sport, Université de Poitiers, Poitiers, France

**Keywords:** social relations, quality of life, physical activities, cognitive activities, wellbeing, aging

## Abstract

**Introduction:**

This review identified and examined the research literature on the effect of participating in physical and/or cognitive activities on older people’s quality of life, to establish whether the social relationships fostered by these activities can be a vector of better physical, mental and social quality of life.

**Method:**

A systematic review of the literature was conducted according to the Preferred Reporting Items for Systematic Reviews and Meta-Analyses (PRISMA) guidelines. We searched four databases (MEDLINE, APA PsycArticles/PsycInfo, PubMed, and Web of Science) for articles published between 1975 and 2022 using search terms related to psychosocial, population, and intervention topics. Studies were eligible if they involved older adults, participation in at least one activity (physical or cognitive), and at least one quality of life related outcome measure.

**Results:**

We selected 20 articles published between 1990 and 2021, the majority concerning studies conducted in English-speaking countries. Ten studies were interventional (introduction of program of activities), and 10 studies were observational (60% quantitative, 40% qualitative). Overall, results revealed a positive impact of the activities on every aspect of quality of life (i.e., cognitive, physical, social, psychological, and quality of life in general).

**Conclusion:**

The present review confirmed the beneficial impact of practicing physical and/or cognitive group activities on older people’s quality of life, but the contribution of social factors and social relationships remains underestimated and not well defined in researches.

## Introduction

The world population is aging, and the number of people aged 60 years or over is expected to double by 2050 [World Health Organization (WHO), 2017]. Aging brings with it a gradual decline in sensory, physical and cognitive abilities. Cognitive decline has been identified as one of the main threats to the health and autonomy in aging (Reuter-Lorenz and Park, 2014). These negative effects raise many issues for older people, surrounding autonomy, aging in place and, more generally, quality of life. *Successful* or *healthy* aging, referred to as *well-aging*, which is defined in the literature as the maintenance of functional autonomy through the optimization of physical, mental and social wellbeing, is thus becoming a major societal challenge (Depp and Jeste, 2006; Gangbè and Ducharme, 2006). However, successful aging remains a complex concept that needs to be better defined in order to promote active, healthy aging for all older people (Estebsari et al., 2020). Well-being is a subjective phenomenon that involves both cognitive components (e.g., life satisfaction) and emotional components (e.g., the balance between positive and negative affect; Diener et al., 1999). The concept of well-being is strongly associated with the more integrative concept of quality of life. The World Health Organization [World Health Organization (WHO), 1994] defines *quality of life* as “a person’s perception of his/her position in life within the context of the culture and value systems in which he/she lives and in relation to his/her goals, expectations, standards, and concerns. It is a broad-ranging concept incorporating, in a complex way, the person’s physical health, psychological state, level of independence, social relationships, personal beliefs, and relationship to salient features of the environment” [World Health Organization (WHO), 1994, p. 29]. This definition highlights the essentially subjective and multidimensional (physical, psychological, social, etc.) concept of quality of life. According to the Ottawa Charter (WHO, 1986), health is defined as “a state of complete physical, mental and social well-being.” Quality of life is therefore a general concept that depends on health, although it is not limited to it. Indeed, some people whose health is considered degraded have a high quality of life, or vice versa (Rejeski and Mihalko, 2001).

Several studies have sought to identify factors that can contribute to successful aging by preserving cognitive health and improving the overall quality of life of older adults. So far, they have highlighted the beneficial effects of cognitive exercise (Kelly et al., 2014; Mewborn et al., 2017; Basak et al., 2020), physical activity (Colcombe et al., 2003; Mandolesi et al., 2018; Carta et al., 2021), training sessions combining cognitive and physical stimulation (Kraft, 2012; Zhu et al., 2016; Gheysen et al., 2018; Guo et al., 2020), and social interactions (Seeman et al., 2001; Pitkala et al., 2011; Kelly et al., 2017; Marsillas et al., 2017). The literature shows that an active and socially engaged lifestyle is linked to better cognitive function in aging, and self-esteem is an important characteristic of wellbeing arising from physical activity (Small et al., 2012; Litwin and Stoeckel, 2016; Yang et al., 2016).

However, although these different factors are beginning to be given greater salience in the literature as means of avoiding cognitive decline and improving quality of life, the health benefits of social relationships continue to be underplayed. Haslam et al. (2005, 2018) maintained that social support and social integration are both highly protective against mortality, and their importance is comparable or even superior to that of many behavioral risks such as smoking, alcohol consumption, and stress. There is considerable evidence that being cut off from social contact with friends, family and other social groups can have extremely negative health consequences, and even lead to premature death (Jetten et al., 2012). By contrast, belonging to and identifying with community groups have been shown to positively predict health and quality of life (McNamara et al., 2013; Haslam et al., 2018; Fong et al., 2019). This idea refers to the notion of *social cure*, which highlights the way in which a person’s social relationships, social networks, social support, and social identity contribute to health outcomes (Jetten et al., 2012; Haslam et al., 2014; Kellezi et al., 2019). This approach states that identification with and sense of belonging to social groups (family, community, sports group, etc.) have an impact on health, social life and wellbeing, leading to improvements in social life and quality of life through the reduction in loneliness, improvement in self-esteem, and perception of available social support (Greenaway et al., 2015; Sani et al., 2015; Kellezi et al., 2019).

The objective of the present literature review was to establish whether the social relationships fostered by some physical and/or cognitive activities can be a vector of better physical, cognitive, psychological and social quality of life for the older people.

## Materials and methods

### Information sources and research process

In order to answer this objective, we conducted a literature review, searching four databases (MEDLINE, APA PsycArticles/PsycInfo, PubMed, and Web of Science) for articles published between 1975 and 2022 Search terms covered the following four topics: (1) social relationships (psychosocial terms), (2) older people (population terms), (3) activity/leisure (intervention terms), and (4) quality of life (health terms).

The keyword search was based on the MEDLINE algorithm. We defined the following list of keywords: (social link or social relationships or social activities) AND (elderly or seniors or aging) AND (physical activities or cognitive activities or physical exercises) AND (well-being or quality of life or mental-physical-psychological health). We applied the same algorithm in the Web of Science, APA PsycArticles/PsycInfo, and PubMed databases. We also conducted a free search, consisting of additional manual searches were undertaken across the first pages of results from the generic web search engine Google Scholar. Moreover, additional searches were carried out by examining the references cited in the included studies and considered to be of interest. We merged these searches and included them in a library we created in Zotero. Duplicates were identified and deleted. In order to make our final selection of articles, we subjected the abstracts to detailed analysis, applying the eligibility criteria (inclusion and exclusion). We assessed point by point whether all the inclusion criteria had been met, and whether the abstracts did not present any exclusion criteria. Abstracts that did not meet all these criteria were excluded from the final selection of articles.

### Eligibility criteria/study selection

The inclusion criteria were as follows:

- Studies in which outcomes (i.e., the dependent variable, quality of life or well-being) are clearly defined will be included.
- Studies in which the independent variable (social relationships) is present in the context of at least one physical and/or cognitive activity will be included.
- The population is defined by the authors as a population of elderly or senior people.

The exclusion criteria were as follows:

- Studies not written in English will be excluded
- Studies that are not journal articles will be excluded (e.g., book chapter, editorial reviews, literature reviews, etc.)
- Studies in which the dependent variable does not concern notion of quality of life or well-being will be excluded
- Studies in which the independent variable (social relationships) is not present in the context of physical and/or cognitive activities will be excluded.
- Studies of populations not defined by the authors as older people’s or elderly or seniors will be excluded.
- Studies conducted on older people who do not live in the community will be excluded. Studies of older populations with pathologies (stroke, Alzheimer’s disease, etc.) will be excluded.
- Studies concerning the evaluation of a measurement tool (e.g., validation of a test or questionnaire) will be excluded.

### Data collection process

The review was not pre-registered, but followed the Preferred Reporting Items for Systematic Reviews and Meta-Analyses (PRISMA) reporting guidelines (Moher et al., 2009), which ensure the transparent and complete reporting of systematic reviews. The articles were selected by the first author (TG) and this selection was then checked independently by all the other authors (DC, GB, CE). Any uncertainty and disagreement between evaluators were resolved by discussion and consensus within the author team (TG, DC, GB, and CE). The first author (TG) extracted the following information: first authors, year of publication, journal, study country, study population, study population size, method, data collection, dependent variables, summary of results.

### Assessing the risk of bias

One of the review authors (TG) assessed independently the methodological quality of the studies included using the modified version of the Downs and Black checklist for assessing randomized controlled trials (RCT) and non-randomized controlled trials (NRCT; Downs and Black, 1998). It consists of 27 items distributed across five subscales: reporting (10 items), external validity (3 items), internal validity bias (7 items), internal validity confounding (6 items), and power (1 item). In the modified instrument, answers are scored 0 or 1, except for one item in the reporting subscale, which is scored 0 to 2. The total maximum score is 28 with higher scores indicating better study quality.

## Results

### Study selection

**Figure 1:**
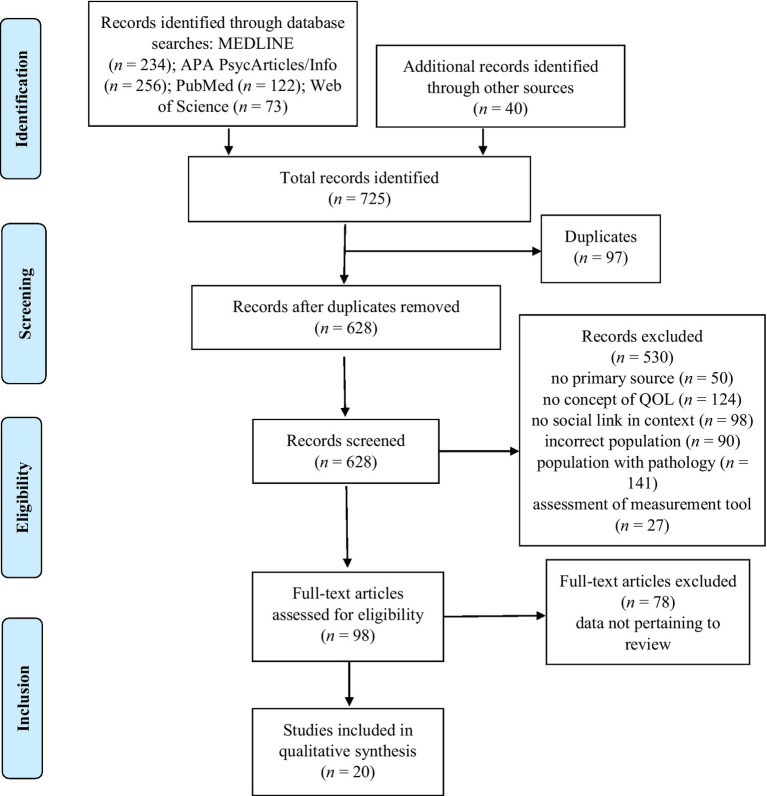
PRISMA flow diagram.

The study selection procedure is illustrated in Figure 1. The free search yielded 40 articles. The MEDLINE keyword search yielded 234 results. Keyword searching with the same algorithm yielded 256 results in APA PsycArticles/Info, 122 in PubMed, and 73 in Web of Science. After removing duplicates, 628 articles were removed based on the title and/or abstract because they did not meet criteria for inclusion, Finally, after reading and analyzing the 98 articles in their entirety according to the eligibility criteria, 20 studies were included in the review.

### Study characteristics

The characteristics of the studies are set out in Table 1. All 20 selected articles were published between 1990 and 2022 (only one before 2000). Eleven articles concerned English-speaking countries (87.5% United States), five Asia, two Africa, and two Europe. The quality of life dimensions they assessed were general quality of life (23%), cognitive (19%), physical (28%), psychological (62%), and social (52%). The studies included have very large population criteria, ranging from 50 to 111 years of age, depending on the study. However, all the studies included in this review have a population whose mean age was over 60 (The lowest mean age was 64.62 in the study by Chang et al., 2014), despite their very large age samples, and according to the World Health Organization, 60 is the age at which people become elderly.

**Table 1:** Characteristics of studies included in review.

Authors	Journal	Study year	Country	Sample size	Mean age in years (*SD*)	% Women	Intervention	Duration of intervention	Quality score
Aday et al.	Journal of Women & Aging	2006	United States	415 women aged 50+ years, 274 living alone and 141 with a spouse	74.4	100%	Multipurpose center for seniors (health, education, recreation, volunteering, social interaction, etc.)		
Bailey & McLaren	Aging & Mental Health	2005	Australia	194 seniors aged 60+ years	Men 69.59 (6.64) women 68.21 (7.78)	55.1%	Physical activity alone or in a group		
Camic et al.	Aging & Mental Health	2014	United Kingdom	24 seniors aged 55+ years	78.3 (8.8)	NA	Painting gallery (observation + creation of artworks)	8 weekly 2-h sessions over 8 weeks	Total score: 12 Reporting: 7 External validity: 0 Internal validity bias: 5 Internal validity: 0 Power: 0
Chang et al.	Health Psychology	2014	United States	2,965 seniors aged 50+ years	64.62 (9.92)	50.2%	Participation in social, mental, physical and productive activities		
Dare et al.	Health and Social Care	2018	Australia	35 seniors aged 60+ years	71	71.4%	2 physical activity groups / 1 painting group / 1 senior center group		
Emery & Gatz	The Gerontologist	1990	United States	48 seniors aged 60+ years	72 (6)	83.3%	Physical exercise program or social activity (card game, art activity, etc.) or waitlisted witness	12-week intervention +12-week exercise program	Total score: 12 Reporting: 6 External validity: 0 Internal validity bias: 5 Internal validity: 1 Power: 0
Gagliardi et al.	Health and Social Care	2019	Italy	73 seniors aged 65+ years	72.88 (8.61)	63%	Intergenerational events held at six farms	Every week for 1 year	Total score: 10 Reporting: 6 External validity: 0 Internal validity bias: 4 Internal validity: 0 Power: 0
Gyasi	Archives of Gerontology and Geriatrics	2019	Kenya	1,200 seniors aged 50+ years	66 (11.6)	63%	Frequency of participation in physical and social activities		
Kamegaya et al.	Psychogeriatrics	2014	Japan	43 seniors aged 65+ years	74.9 (5.9)	90%	Intervention = physical activity program (stretching, strength training, aerobics, balance) + leisure (cooking, crafts, competitive play) Control = no program	12-week intervention with 2 h per week in a community center	Total score: 14 Reporting: 7 External validity: 0 Internal validity bias: 5 Internal validity: 2 Power: 0
Kanamori et al.	Scientific Reports	2016	Japan	21,684 seniors aged 65+ years	73.5 (6)	52.1%	Physical activity alone or with others		
Kim et al.	Health Care for Women International	2015	Korea	11 women aged 68+ years	Range: 68–81	100%	Participation in activities at a senior center (yoga, recreational program, regular exercise, sports, traditional Korean games)		
Kim et al.	International Journal of Qualitative Studies on Health and Well-being	2014	Korea	10 seniors aged 65+	71	70%	Membership of a sports club for many years		
Komatsu et al.	BMC Geriatrics	2017	Japan	26 seniors aged 60+ years	74.69 (range: 66–86)	57%	Participation in regular group exercises in the community (Fujisawa+10 program)		
Levinger et al.	BMC Geriatrics	2020	Australia	80 seniors aged 60+ years	73 (7.4)	82.1%	Exercise program in an exercise park (strength, balance, coordination, etc.)	12-week intervention 80 min twice a week, then 6 months unsupervised independent access to the park or access to supervised exercise sessions, but no group activity.	Total score: 12 Reporting: 8 External validity: 0 Internal validity bias: 3 Internal validity: 1 Power: 0
MacAulay et al.	Psychomusicology: Music, Mind, and Brain	2019	United States	35 seniors aged 55+ years	70 (5.12)	98%	Music (recorder playing) project (Maine Understanding Sensory Integration and Cognition Project)	12 1-h group sessions	Total score: 10 Reporting: 7 External validity: 0 Internal validity bias: 3 Internal validity: 0 Power: 0
McAuley et al.	Psychology and Health	2000	United States	80 seniors aged 60–75 years	65.5	77.5%	Aerobic physical activity (walking) program or stretching and toning program, practiced alone or in a group	Three 1-h sessions per week for 6 months	Total score: 15 Reporting: 7 External validity: 0 Internal validity bias: 6 Internal validity: 2 Power: 0
Meeks et al.	Aging and Mental Health	2020	United States	42 seniors aged 60+ years	66.13 (4.61)	63.6%	Theatergoing (seven plays)	Seven plays over two consecutive seasons	Total score: 11 Reporting: 7 External validity: 0 Internal validity bias: 4 Internal validity: 0 Power: 0
Nadasen	Journal of Women & Aging	2008	South Africa	30 seniors aged 60+ years	69.6 (7.6)	100%	Line dance program	12 months	Total score: 9 Reporting: 5 External validity: 0 Internal validity bias: 4 Internal validity: 0 Power: 0
Pentikäinen	PLOS ONE	2021	Finland	162 seniors aged 60+ years	High singer: 72.8 (5.7) Low singer: 69.2 (4.5) Control: 70.2 (6.5)	High-level singer: 79.1% Low-level singer: 65.5% Control: 85.7%	Follow-up of elderly choir singers and non-choir singers		
Yuen et al.	Occupational Therapy International	2011	United States	12 seniors aged 60+ years	75.3 (8.5)	91%	SAASY Program (theater instruction and experience)	Weekly 2-h sessions over 6 weeks + participation in five performances + giving four performances 2 months after the end of the 6 weeks	Total score: 10 Reporting: 6 External validity: 0 Internal validity bias: 4 Internal validity: 0 Power: 0

NA: not available.

### Methodologies

Of the 20 studies, 10 were interventional, involving a program of leisure, physical or cognitive activities, with a pre−/posttest analysis plan, and 10 were observational (60% quantitative, 40% qualitative). The quantitative studies were cross-sectional, and the qualitative studies were based on focus groups or interviews. Eight of the 20 studies compared the effects of activity according to group, while the other 12 studies assessed the effects of activity with no group comparison.

### Study rigor and study quality

The summary of the quality scores is displayed in Table 1. The quality scoring descriptive statistics include a mean of 11,5. The quality assessment of the 10 interventional studies highlights that the majority of them were of low quality. The scores were very low in external validity, internal validity - confounding and statistical power. This indicates that the studies had the insufficient statistical power to detect important effects and none of the findings could be generalized to older population. Serious risk bias can be highlighted such as small samples, no comparison or control group, and lack of details regarding the intervention.

### Outcomes

The results of the studies included in the review are set out in Table 2. More than half (52%) of the studies looked at the effects of participating in physical activities (sports clubs, aerobic activity programs, etc.) on quality of life, 42% were interested in the effects of participating in cognitive and/or social activities (day center for seniors, card games, social farm, etc.) and 38% looked at the impact of leisure activities (theater, musical training, etc.).

**Table 2:** Results of Studies Included in Review.

Author	Objective and method assessment times	Quality of life variable (cognitive, physical, psychological, social)	Data collection	Summary statistics	Results
Aday et al. (2006)	Examine influences of late-life friendships and senior center activities on aging women’s health and wellbeing Quantitative: questionnaires	Social support network Perceived independence Mental health Depressive symptoms Life satisfaction	Series of open-ended questions on life satisfaction, emergence of a social network, strength of that social network, perceived and actual emotional and instrumental support Depressive symptoms were assessed using a short version of the Geriatric Depression Scale Participation measured via responses to a checklist of activities and programs conducted at the centers	Independent samples t-tests Bivariate correlations Comments stressing is provided for illustrative purposes.	Although women in couples and single women attended the centers with the same frequency, single women spent more time per visit at the center and were significantly more likely to participate in the various programs available (education, health promotion, etc.) No difference between single women and women in couples on self-rated health status Single women were significantly more likely to feel less lonely, laugh more, worry less about the future, have more energy, and handle stress better as a result of attending the center Few differences emerged between single women and women in couples in terms of the formation and development of friendships, perceived support, and emotional depth of friendships 89.2% of single women reported that they had developed close friendships since going to the senior center. The high level of social support evidenced shows that 84.2% of those surveyed felt that the friends they had made could help them get by if they were in need Women who found social support at the center were more likely to engage in activities there
Bailey and McLaren (2005)	Determine whether physical activity alone or combined with other types of activity predicts depressive symptoms, which in turn predicts suicidal ideation Quantitative: questionnaires	Sense of belonging (psychological state and history) Depressive symptoms Suicidal thoughts	Yale Physical Activity Survey assessing level of physical activity (duration, type of activity (alone or in group), etc.) and Sense of Belonging Instrument Suicide subscale of General Health Questionnaire Zung Self-Rating Depression Scale	Correlation analysis	Negative correlation between physical activity practiced with others and depressive symptoms (more hours of group practice associated with fewer depressive symptoms) Positive correlation between the two aspects of sense of belonging and physical activity practiced with others (higher scores on sense of belonging, psychological status, and background associated with more physical activity time with others) These higher sense of belonging scores were associated with lower levels of depressive symptoms and suicidal ideation
Camic et al. (2014)	Examine possible impact of a longer intervention involving observation and art-making sessions in a gallery on social inclusion, quality of life, and activities of daily living among people with disabilities and their caregivers Mixed quantitative and qualitative: questionnaire + interview Pre- and postassessments	Health-related quality of life	Dementia Quality of Life Measure-4 assessing health-related quality of life in individuals with dementia Bristol Activities of Daily Living Scale assessing ability of individuals with a disability to undertake activities of daily living Semistructured interviews	Parametric and non-parametric statistical tests Interview and observational data were analysed using thematic analysis	Persons with disabilities exhibited a higher level of cognitive engagement, during both art observation and the making of art, as well as outside of the group No significant difference between quality of life scores before and after the intervention: self-reported quality of life of people with disabilities did not improve, but remained stable during the intervention “Making art, creating artwork, and occupying oneself are all good, but it is not very easy to do sitting at home. So, getting together with a group of people to interact like this is what makes it a success, I think” The art gallery was defined as a special and empowering environment that helped participants feel active members of society, socially included and valued as individuals: The gallery was a nice, quiet place where the brain could work
Chang et al. (2014)	Examine relationship between social relationships and health among seniors, looking at whether leisure plays a mediating role in the association between social relationships and health outcomes Quantitative: questionnaire + rating scale	Social relationship Physical health Psychological wellbeing	Social relationships, level of social support assessed by three questions (e.g., “How often do we make too many demands on you?”) rated on 3-point scale Questions about frequency of leisure activities rated on 6-point scale for 18 distinct leisure activities divided into four types of activities (mental/social/physical/productive) Physical health: self-reported Psychological wellbeing, depressive symptoms assessed with Center for Epidemiologic Studies Depression Scale Life satisfaction as assessed with Diener scale Insomnia assessed with four questions (yes/no) on sleep quality	Analyses were performed using structural equation modeling (SEM)	Significant direct effects between: social relationships and leisure activities social relationships and psychological wellbeing social relationships and physical health leisure activities and psychological wellbeing leisure activities and physical health Links between social relationships and physical health or psychological wellbeing were strengthened by the presence of leisure activity A main effect model, in which higher quality social relationships may motivate the adoption of health-promoting behaviors such as leisure activities and bring greater health benefits. Social networks can value and encourage participation in leisure activities as a way to maintain health
Dare et al. (2018)	Identify the facilitators of and barriers to community participation, looking at whether they differ between those who regularly participate in community activities, those who participate only irregularly, and those who do not participate at all. Qualitative: focus group or individual interview	Social facilitators of and barriers to participation in community activities	Focus group and interviews, transcribed and coded	The detailed notes and memos from the focus groups and interviews were analysed by an inductive process of thematic analysis	Friendship and function: Respondents valued the opportunity to socialize with others and derived more enjoyment from their interactions than from the activity itself / The ability to connect with others was particularly important for respondents living alone / One woman referred to her craft group as a “free support group,” while a single woman noted that she would be “pretty socially isolated” if she did not attend her craft group. However, one woman who participated irregularly described her experience as being “alone in a crowd” While interest in an activity may motivate people to join a group, it is the sense of belonging and connection generated by the group that maintains participation and social engagement Availability and accessibility: problem related to the availability of programs (location, timing, structure) Competing responsibilities and priorities: family engagement (care of grandchildren), professional engagement Changing of the guard: declining enrollment is seen as a threat to the sustainability of senior programs
Emery and Gatz (1990)	Study the effects of a 12-week physical exercise program on measures of cognitive functioning and psychological wellbeing in community-dwelling older adults Quantitative: questionnaires + physical test Pre- and postintervention assessments	Physical function Psychological wellbeing (anxiety, depressive symptoms, etc.) Cognitive function	Physical fitness assessed with step test and sit-stand test, with measurement of heart rate, systolic and diastolic blood pressure For psychological wellbeing, mood assessed with Center for Epidemiologic Studies Depression Scale, and anxiety with the Center for Epidemiologic Studies Anxiety Scale Assessment of locus of control and mastery using Rotter’s Internal-External Scale, Pearlin Mastery Scale, and Lau’s health-specific locus of control questionnaire Cognitive functioning assessed with the WAIS-R Digit Symbol test, and verbal and working memory with a writing speed test	MANOVA: 2 × 2 (Time x Group) repeated measures multivariate analysis of variance Correlational analyses	Participants in the control group had higher levels of self-rated happiness at both timepoints Overall, the exercise program did not appear to contribute significantly to physiological functioning or psychological well-being No significant main effects or interactions for cognitive function. Overall, there was very little change on cognitive measures in either group No significant physical improvement (flexibility, fitness) No significant effect of exercise program on depressive symptoms or anxiety, so no further evidence of another association between improved control or locus of control and decreased depressive symptoms or anxiety
Gagliardi et al. (2019)	Assess a 1-year social farming program (horticultural and professional activities) aimed at older adults in good general health, conducted between 2014 and 2015 at six farms. Determine whether participating in social farming activities promotes quality of life, social relationships, leisure participation, and physical activity for older adults. Quantitative: questionnaires Pre- and postintervention assessments	Quality of life Social relationships	Quality of life was assessed with the WHOQOL-AGE (physical and mental health, sense of inclusion, satisfaction with quality of life) Social relationships and participation in activities were assessed by the frequency and number of leisure activities usually practiced, selected from a list Physical activity was assessed with the Minimum Data Set for Home Care	Independent samples Student’s t test for continuous variables and the chi-square test for categorical variables.	Positive changes were observed in social participation measures Contact with friends or family members at least once a week increased significantly Social farms lead to the expansion of social networks, feelings of solidarity, and an improvement in the environment Increased number of leisure activities No significant difference in quality of life between T0 and T1
Gyasi (2019)	Investigate the effects of regular physical activity, social support, and the interaction between the two on psychological distress outcomes in community-dwelling older adults Quantitative: questionnaires	Psychological distress	Physical activity assessed with Global Physical Activity Questionnaire Psychological distress assessed with 10-item Kessler Psychological Distress Scale Social support assessed by measuring frequency of contact with family or friends and frequency of social participation (physical/leisure activity, civic/social organization)	Bivariate (unadjusted) regression analysis Multivariate logistic regression models	Results showed that both regular physical exercise and social support were strongly negatively associated with psychological distress Social support was found to have a moderating effect on the relationship between physical activity and psychological distress, indicating that increasing social support strengthened this negative association
Kamegaya et al. (2014)	Explore effectiveness of a comprehensive program of physical and recreational activities in preventing cognitive decline in older people Quantitative: questionnaires + tests Pre- and postintervention assessments	Cognitive function Physical function Functional capacity Level of social support Depressive symptoms	Cognitive function: five cognitive tests (attention, memory, visuospatial function, language, and reasoning) + symbol substitution test (executive function) Physical function: grip strength, timed acceleration, 5-m walk test, and reach test Social support level: Lubben scale (isolation + perceived social support) Functional capacity: Tokyo Metropolitan Institute of Gerontology Competency Index 20 (satisfaction with daily life) Depressive symptoms: Global Physical Activity Questionnaire	Chi-square test conducted in categorical data Two-sample t-tests were conducted in continuous variables repeated measures ANCOVA	Significant increase in scores on cognitive test in intervention group compared with control group (on animal naming and analogy tasks), but no difference on the other cognitive test scores (executive function, attention, memory, visuospatial function) Significant improvement in quality of life in intervention group, but not in control group No other significant differences observed (neither subjective health status, social support, functional ability, nor depressive symptoms) No change in physical function
Kanamori et al. (2016)	Examine whether the association between subjective health status and exercise differs according to whether the exercise is performed alone and/or with others, adjusting for exercise frequency Quantitative: rating scale	Health status Sociability	Health status assessed with questions such as “How is your current health?” Assessment of exercise alone or with others: “How often do you exercise alone?,” “How often do you exercise with a relative, friend or acquaintance?” Sociability assessed by measuring frequency of meeting friends, receiving and providing instrumental support, receiving and providing emotional support (yes/no questions)	Multivariable logistic regression	Self-rated poor health was significantly lower for all exercise groups than for nonathletes Self-rated poor health was significantly lower for those who exercised both alone and with others, and for those who only exercised with others compared with those who only exercised alone Increased frequency of exercise with others had significant health benefits Although both exercise alone and exercise with others appeared to have health benefits, social relationships may be the mechanism that subtends the health benefits of exercising with others
Kim et al. (2015)	Explore benefits of social engagement in leisure activities among older Korean women Qualitative: semistructured interview	Development of social relationships Psychological wellbeing Physical health	Assessment of participants’ thoughts, feelings, knowledge, attitudes, and experiences about leisure activities through interviews Sample question: “Tell me about the kinds of things you do for fun in your free time.”	The data were coded and analyzed using the constant comparative method suggested by Merriam (1998) A three-step analysis consisting of (a) open coding, (b) axial coding, and (c) selective coding	Becoming involved increased older people’s opportunities to interact with others, expand their social networks, and share their feelings and emotions. Leisure activities can be used as a way of fostering close relationships Leisure activities provided a context for participants to actively reduce their negative feelings and emotions whilst increasing their self-esteem and self-confidence, which ultimately improved their quality of life Significant improvement in all measures of physical function (e.g., muscle strength, flexibility, motor skills, coordination, mobility, pain reduction)
Kim et al. (2014)	Explore benefits of serious engagement in leisure activities at a sports club among older Korean adults Qualitative: interview	Overall benefit of engaging in leisure activities	Assessed by interview (e.g., “Tell us about your overall experience as a club member,” “What benefits do you feel when you participate in these activities?”)	Three-step data analysis: (1) the creation of the open coding, (2) the generation of axial coding, and (3) selective coding through an interconnected storyline.	Main benefits of leisure activities: Psychological benefits (fun, positive feelings, improved self-esteem and confidence) Creation of social support (fostering of positive social interactions, development of sense of friendship) Improved physical health (increased physical strength and endurance)
Komatsu et al. (2017)	Explore experiences of older adults engaging in regular group exercise and study their perceptions of the physical, mental, and social changes they experience as a result Qualitative: semistructured interview	Physical, mental and social changes	Assessed by interview (e.g., “What change have you noticed in your body, memory, activity or daily life as a result of participating and continuing in the program?,” “Have you perceived any changes in yourself affecting your interpersonal relationships or social interactions?”)	Constant comparative method in the grounded theory approach	Group exercise contributed to participants’ physical, mental and social wellbeing and helped them improve or maintain their functional health, socialize with their peers, and enjoy life. - They felt socially connected, full members of society, and safe in the community Participation in group exercises resulted in interaction with others, prevention of isolation by socializing with others, and stimulation in their daily lives Participants felt that the stimulation of talking to people before and after exercise were good for their mental health. They believed that keeping their minds active and talking to peers helped maintain their cognitive function Participants became more active and began to enjoy their lives more. The group exercise provided an opportunity not only for physical activity, but also for socializing
Levinger et al. (2020)	Assess effects of supported physical activity program on physical, mental, social and health outcomes through use of Senior Exercise Park physical activity program for older adults Quantitative: questionnaires + self-reported measurements and physical tests Assessments at baseline, 3 months, and 9 months	Physical function Mental well-being Depressive symptoms Psychosocial outcomes (loneliness, social isolation)	Physical activity assessed with self-report measurement of caloric expenditure (CHAMPS) and physical tests (chair lift, TM 2 min, TM 4 m, step test); risk of falling assessed with Falls Risk for Older People in the Community Psychosocial: assessment of health-related quality of life with 5Q-5D-5L (mobility, self-care, usual activities, pain/discomfort, and anxiety/depressive symptoms), pleasure with Physical Activity Enjoyment Scale, social isolation with the Lubben Social Network Scale, fear of falling with the Falls Efficacy Scale International, and loneliness with the loneliness scale Mental health, assessment of wellbeing with Global Physical Activity Questionnaire	Repeated measures analysis of variance (ANOVA)	Significant increase in level of physical activity after intervention Significant improvements in all measures of physical function between baseline and 9-month follow-up Significant improvements in all measures of physical function, quality of life, wellbeing, fear of falling, depressive symptoms and loneliness No change in socialization (social isolation) and self-efficacy for exercise outcomes Significant changes were only observed in the areas of health-related quality of life, mobility, and self-care No changes were observed for remaining health-related quality of life measures (usual activity, pain/discomfort), other than significant reductions in fear of falling and risk of falling
MacAulay et al. (2019)	Study effect of music training on social, emotional and cognitive functions in older adults Mixed method: quantitative (cognitive test + questionnaires) and qualitative (semistructured interview) Pre- and postintervention assessments	Mental health General cognitive function	Cognitive measures: episodic memory, processing speed, executive functions with Uniform Data Set, Version 3 Neuropsychological Battery and Montreal Cognitive Assessment Mental health (social and emotional wellbeing) assessed with semistructured interviews	Repeated-measure analyses of variance and Wilcoxon signed-ranks test	Improvement in executive attention and processing speed, and trend toward improvement in working memory Significant change in global cognition, verbal fluency, and visual memory performance after music intervention-improvements in motor coordination and memory Development of valuable socialization and sense of accomplishment, building self-esteem and confidence Music group reported reduced stress and improved emotional wellbeing Learning, social engagement, and social support were top three benefits of group participation
McAuley et al. (2000)	Determine whether different exercise environments influence affective responses independently of the many effects of exercise intensity and duration Examine extent to which changes in exercise self-efficacy are associated with changes in affective responses to acute exercise independently of exercise dose Quantitative: questionnaires + physical tests Pre- and postintervention assessments	Self-efficacy Affects	Mental status assessed with Pfeiffer Mental Status Questionnaire Self-efficacy assessed on confidence scale (participants’ belief in their physical ability to successfully complete 5-min walks) Affects (positive wellbeing, psychological distress, and fatigue) assessed with Subjective Exercise Experiences Scale	Latent growth curve methodology (LGM)	The group environment seemed to have a more beneficial effect on older people’s wellbeing than the individual environment did, even when authors controlled for the intensity and duration of the exercise Changes in self-efficacy were significantly related to changes in positive wellbeing, psychological distress, and fatigue, but in different ways across the three conditions For the mild and only group: a greater increase in self-efficacy was associated with increased wellbeing and less psychological distress. Changes in efficacy were not related to changes in affect in moderate group condition The social composition of the exercise environment influenced affective responses Organized group exercise sessions were an opportunity for the development of social support
Meeks et al. (2020)	Follow a cohort of older theatergoers for two seasons to examine their experience during each play they attend Quantitative: questionnaires Pre- and postintervention assessments	Affects Sense of belonging Satisfaction and pleasure in watching performance Wellbeing	Assessment of psychological and social wellbeing using Ryff scale Affect assessed with Positive and Negative Affect Scale and Bradburn Affect Scale Social engagement and sense of belonging assessed by participants (indicating people with whom they attended the performances and how close and connected they felt to these people) Extent of knowledge of the play and the pleasure it brought	Random coefficient models Multiple regression models	Social engagement, pleasure, knowledge, and sense of belonging contributed significantly to positive affect over time Neither belonging nor social engagement contributed significantly to wellbeing when the initial wellbeing values were considered Belonging and social engagement had indirect effects on wellbeing through their impact on positive affect
Nadasen (2008)	Examine how participants in dance classes are directly affected by their line dancing activities Qualitative: interview and open-ended questions Pre- and postintervention assessments	Socialization	Assessment through individual or group interviews and use of open-ended questions such as “What attracted you to line dancing?,” “In addition to regular dance classes, do you participate in other activities as a direct result of being a member of this group?”	Open coding process Thematic content analysis The methodology of this study was qualitative and followed Ingrisch’s (1995) notion	Line dancing met two needs: have fun and feel independent. Belonging to a group made it possible to engage collectively in activities that benefit everyone Participation in various other activities cemented and strengthened their friendship, expressed through exchange of emotional and social support The additional activities (costume making, participation in competitions, etc.) opened up new perspectives for expression and opportunities for self-discovery Women over 70 years generally admitted that their lives would be extremely lonely and unsatisfying without dance
Pentikäinen et al. (2021)	Explore whether active participation in choral singing is associated with cognitive, emotional and social wellbeing and quality of life in healthy older adults Quantitative: cognitive tests + questionnaires	General cognitive function Social wellbeing Depressive symptoms General quality of life	General cognition was assessed with Montreal Cognitive Assessment Executive functions were assessed with the Phonemic Fluency Test, Trail Making Test, FAT, Simon Task, Digit Symbol Substitution Test and Digit Span Test of the Wechsler Adult Intelligence Scale IV, and Corsi Block Test Episodic memory was assessed with the Wechsler Memory Scale III Verbal skills were assessed with the WAIS-IV vocabulary test and semantic fluency task Cognitive functioning was assessed with the Cognitive Failure Questionnaire and Prospective and Retrospective Memory Questionnaire Depressive symptoms with the Center for Epidemiologic Studies Depression Scale Social wellbeing with the Social Provisions Scale Quality of life with the WHOQOL-Bref Role of music in daily life by the Music Engagement Questionnaire Levels of cognitive and physical activity assessed with the LEQ	Independent-samples t tests One-way ANOVAs Chi-square tests Univariate ANCOVAs	Choir singers performed significantly better than controls on the verbal flexibility subdomain of executive functions. No significant differences between groups in other cognitive domains (inhibition, memorization, processing speed, episodic memory, etc.) For social wellbeing (Social Provisions Scale), scores were higher for high-activity choral singers than for low-activity choral singers and controls, while low-activity choral singers and controls did not differ WHOQOL-Bref general health scores were higher for low-activity choral singers than for high-activity choral singers and controls, but did not differ between high-activity choral singers and controls MusEQ scores were higher in both high- and low-activity choral singers, compared with controls There were no significant effects for depressive symptoms
Yuen et al. (2011)	Assess the impact of participation in the SAASY program on individuals’ psychological wellbeing and health-related quality of life Qualitative and quantitative: interview and questionnaire Pre- and postintervention assessments	General wellbeing (anxiety, depressive symptoms, vitality, etc.) Physical functioning Mental health General health Social functioning	Interview (e.g., “How would you describe your health status before the program?,” “How would you describe your health status since starting the program?”) General wellbeing (depressive symptoms, anxiety, self-control, vitality, etc.) assessed with General Well-Being Schedule Psychological wellbeing (physical role, emotional role, mental health, social functioning, etc.) assessed with SF-36	A one-sided, non-parametric Wilcoxon signed-rank test Interview transcripts: an investigator triangulation approach was used in cross-checking and verifying the interpretation of data	Significant improvement in wellbeing as measured with General Well-Being Schedule and in physical health after the program No significant improvement in SF-36 score Participants mentioned social benefits of drama classes: opportunity for social interaction, camaraderie, friendship Increased sense of self-confidence, self-advocacy

### Presentation of results

All the results of the present literature review are presented in the same way: a brief description of the studies concerned, following by a paragraph on the interventional ones and a paragraph on the observational ones. In each case, we report any divergent results: first, the results confirming that physical and/or cognitive activities can be a vector of better physical, cognitive, psychological and social quality of life for the older people; then the results refuting (or at least not validating) this notion.

### Physical quality of life

Six of the 20 studies examined the effects of various activities on physical quality of life. Four of them assessed the effects of an intervention program (Emery and Gatz, 1990; Yuen et al., 2011; Kamegaya et al., 2014; Levinger et al., 2020), and the other two (observational) studies looked at activities already performed by older adults and assessed the benefits of social engagement in terms of physical function (Chang et al., 2014; Kim et al., 2015).

Two of the four interventional studies showed an increase in self-reported physical activity after the intervention (Yuen et al., 2011; Levinger et al., 2020), as well as substantial improvements in all three objective measures of physical function (fall risk, endurance, strength; Levinger et al., 2020). Emery and Gatz (1990) and Kamegaya et al.’s (2014) studies found no improvement in physical function, as measured by an objective assessment of physical ability after participation in a 12-week intervention program.

The two observational studies assessing physical abilities subjectively, via questionnaires or interviews, found improvements in all the physical abilities that were assessed (e.g., muscle strength, flexibility, motor skills, coordination, mobility, pain reduction; Chang et al., 2014; Kim et al., 2015). Chang et al. (2014), highlighted direct links between social relationships and physical health, and between leisure activities and physical health.

### Cognitive quality of life

Four of the 20 studies examined the effects of various activities on cognitive quality of life. Three of them assessed the effects of an intervention program (Emery and Gatz, 1990; Kamegaya et al., 2014; MacAulay et al., 2019), while the fourth (observational) study explored the effect of participating in leisure activities on cognitive functions (Pentikäinen et al., 2021).

One of the three interventional studies highlighted significant changes in global cognition, verbal fluency and visual memory performance after a music program, with improvements in executive attention, processing speed, and working memory (MacAulay et al., 2019). The second study found a significant increase in cognitive scores for the physical activity program and leisure intervention group compared with a control group on an animal naming task and analogy task (Kamegaya et al., 2014). However, there were no other differences on the other cognitive tasks probing executive function, attention, memory, and visuospatial function (Kamegaya et al., 2014). The third interventional study, which assessed the effect of physical exercise program or social activities on cognitive functions, found no effect of the program on any of the cognitive functions (e.g., verbal and working memory, processing speed) in either group in their study (Emery and Gatz, 1990).

The observational study by Pentikäinen et al. (2021) assessed the effects of choir membership on participants’ cognitive abilities. The authors highlighted the benefits of this social group activity on cognitive functions and in particular on the verbal flexibility subdomain of executive functions. However, although they highlighted a significant difference between choir and control groups on the subdomain of verbal flexibility, the groups did not differ on other cognitive domains such as memorization, episodic memory, processing speed, and inhibition (Pentikäinen et al., 2021).

### Social quality of life

Eleven of the 20 studies examined the effects of various activities on the social dimension of quality of life. Six of them assessed the effects of an intervention program (musical training, physical activity program, etc.; McAuley et al., 2000; Nadasen, 2008; Yuen et al., 2011; Kamegaya et al., 2014; MacAulay et al., 2019; Levinger et al., 2020), while the other five (observational studies) explored the advantages and effects of participating in leisure activities on social quality of life (Aday et al., 2006; Chang et al., 2014; Kim et al., 2015; Dare et al., 2018; Pentikäinen et al., 2021).

For the interventional studies, the authors showed that taking part in leisure and social group programs is rewarded by social support (McAuley et al., 2000) and valuable socialization (MacAulay et al., 2019). Social empowerment provides the opportunity for social interactions or relationships (Yuen et al., 2011) that strengthen friendships, expressed through mutual emotional and social support (Nadasen, 2008). MacAulay et al. (2019) concluded that the learning, social engagement, and social support offered by these activities are the direct result of group participation. Moreover, McAuley et al. (2000) explained that the social component of the exercise environment can influence affective responses to exercise. However, although both Kamegaya et al. (2014) and Levinger et al. (2020) agreed that group exercise improves quality of life, they failed to observe any other differences in social support (Kamegaya et al., 2014) or changes in socialization and social isolation (Levinger et al., 2020) as a result of the program. Similarly, Yuen et al. (2011) found no significant improvement in social functioning scores.

The observational studies also reported positive effects of leisure or physical activity on quality of life at the social level. Leisure activities are a means of promoting social relationships (Kim et al., 2015), which in turn foster people’s engagement in these activities (Aday et al., 2006). Some authors have underlined the importance of the social context of practice regarding people’s engagement in these activities and the effects on health, notably at the social level, but also in a more general way. For example, Chang et al. (2014) suggested that there is a link between social relationships and leisure activities, and explained that better social relationships can enhance and motivate the adoption of health-promoting behavior. Dare et al. (2018, p. 879) stated that “While interest in an activity may motivate people to join a group, it is the sense of belonging and connection generated by the group that maintains participation and social engagement.” In Aday et al. (2006)’s study, women who found social support at the center were more likely to engage in center activities. By engaging in social activities, participants can increase their opportunities to interact with others, expand their social networks (Kim et al., 2015), and increase their perceived social wellbeing (Pentikäinen et al., 2021).

Participating in activities gives people an opportunity to develop close friendships and raise the level of perceived social support (Aday et al., 2006). Moreover, Dare et al. (2018) found that respondents lent more importance to the opportunity to socialize with others and the enjoyment of their interactions than to the activity itself. The ability to connect with others was particularly important for people living alone. However, when Aday et al. (2006) examined the influence of friendship at the end of life and participation in center activities on health by comparing single women and women in couples, they found little difference between the two groups in terms of friendship development, perceived social support, and depth of friendships. In general, there was no difference between single women and women in couples in terms of self-rated health (Aday et al., 2006). Finally, Dare et al. (2018) mentioned barriers to social participation such as lack of availability or accessibility, and problems of social integration, citing the testimony of a participant who said she was “alone in a crowd.”

### Psychological quality of life

Thirteen of the 20 studies examined the effects of various activities on psychological quality of life. Seven (Emery and Gatz, 1990; McAuley et al., 2000; Yuen et al., 2011; Kamegaya et al., 2014; MacAulay et al., 2019; Levinger et al., 2020; Meeks et al., 2020) of them assessed the effects of an intervention program (musical training, physical activity program, theater, etc.), while the other six (Bailey and McLaren, 2005; Aday et al., 2006; Chang et al., 2014; Kim et al., 2015; Gyasi, 2019; Pentikäinen et al., 2021) observational studies explored the effects of taking part in social, sports and leisure activities on the psychological dimension of quality of life.

The interventional studies showed that participating in leisure, physical and social group programs improves quality of life in general (Emery and Gatz, 1990; McAuley et al., 2000; Yuen et al., 2011; Kamegaya et al., 2014), and more specifically reduces depressive symptoms and enhance sense of belonging, self-esteem, and self-confidence (McAuley et al., 2000; Yuen et al., 2011; Levinger et al., 2020). McAuley et al. (2000) found a reduction in stress and an improvement in emotional wellbeing after a music training intervention. Meeks et al. (2020) concluded that social engagement, enjoyment, knowledge, and perceived sense of belonging contributed significantly to positive affect. These authors claimed that belonging and social engagement have only indirect effects on quality of life, through their impact on positive affect. McAuley et al. (2000) agreed with Meeks et al. (2020), reporting that changes in self-efficacy during the program were significantly related to changes in positive quality of life, psychological distress, and fatigue. These authors explained that a greater increase in self-efficacy was associated with better quality of life and less psychological distress (McAuley et al., 2000). However, Kamegaya et al. (2014) and Emery and Gatz (1990) reported no difference between their groups and no improvement in depressive symptoms or anxiety. Emery and Gatz (1990) reported in particular that the exercise program did not appear to contribute overall to improvements in psychological wellbeing. Yuen et al. (2011) and Levinger et al. (2020), found no improvement in emotional role scores and no change in self-efficacy for exercise outcomes.

Observational studies explored the effects of participating in various activities on psychological quality of life. Kim et al. (2015) and Pentikäinen et al. (2021) showed that participating in singing or social engagement activities reduces negative feelings and emotions, whilst increasing self-esteem and self-confidence, which ultimately improves quality of life. Pentikäinen et al. (2021) added that general health scores were higher for low-activity singers than for high-activity singers. Three of the six studies found associations between activity and psychological wellbeing. Gyasi (2019) highlighted the links between physical activity, social support, and psychological distress: physical activity and social support were both negatively associated with psychological distress, while social support moderated the relationship between physical activity and psychological distress (i.e., the greater the social support, the stronger the negative association between physical activity and psychological distress). Bailey and McLaren (2005) highlighted the relationship between physical activity and depressive symptoms, reporting that more hours of group physical activity were associated with fewer depressive symptoms. Higher scores on sense of belonging and psychological status were also associated with more hours of group physical activity. These higher levels of sense of belonging were associated with lower levels of depressive symptoms and suicidal ideation (Bailey and McLaren, 2005). Chang et al. (2014) found a direct relation between social relationships and psychological wellbeing, and a direct effect of leisure activities on psychological wellbeing. They showed that the links between social relationships and physical health or psychological wellbeing were strengthened by leisure activity (Chang et al., 2014). For their part, Aday et al. (2006) observed that single women were more likely to feel less lonely, laugh more, worry less about the future, have more energy, and handle stress better as a result of attending a center. However, Pentikäinen et al. (2021) found no effect of song participation on depressive symptoms.

### General quality of life

Five of the 20 studies examined the effects of various activities on general quality of life. Two (Camic et al., 2014; Gagliardi et al., 2019) of them assessed the effects of an intervention program (agricultural or cultural). The other three (observational) studies examined the benefits of engaging in a club’s leisure activities (Kim et al., 2014), as well as changes in participants’ health status following regular group leisure activity (Komatsu et al., 2017), or in comparison with single or group modes of practice (Kanamori et al., 2016).

For the interventional studies, Camic et al. (2014) and Gagliardi et al. (2019) reported an improvement in the level of cognitive engagement (Camic et al., 2014), as well as benefits at the social level. They showed that the intervention setting (art gallery or farm) increased social participation, with increases in participants’ contacts with family or friends, and in the number of leisure activities. In general, they reported that in both settings, the interventions led to the expansion of social networks and a sense of solidarity (Gagliardi et al., 2019), and helped people feel active members of society, socially active, and valued as individuals (Camic et al., 2014). However, the authors found no significant difference between general quality of life scores before and after the program, reporting that self-reported quality of life remained stable across the intervention (Camic et al., 2014; Gagliardi et al., 2019).

For the observational studies, Kim et al. (2014) did not examine changes in quality of life, but rather the perceived benefits of serious engagement in leisure activities. They highlighted three main benefits of this engagement, namely (1) a psychological benefit, with the development of a positive sense of pleasure, improved confidence, and self-esteem, (2) a social benefit, with the creation of social support, the development of positive social interaction fostered in these activities, and the development of a sense of friendship, and (3) a physical benefit, with an improvement in physical health due to the increase in physical strength and endurance (Kim et al., 2014). Kanamori et al. (2016) and Komatsu et al. (2017) were interested in the perception of changes in health according to the context of single or group practice. Kanamori et al. (2016) found that engaging in group physical activity increased self-reported health, as self-reported poor health was significantly lower for all exercise groups, compared with participants who exercised alone. The authors suggested that social relationships are behind the health benefits of group exercise, and regular group exercise has important health benefits. These results are consistent with those of Komatsu et al. (2017), who showed that group exercise contributes to individuals’ physical, mental and social quality of life, by helping them to improve or maintain their functional health, socialize, and enjoy life. They reported the social effects of group exercise, noting that participation in group exercise resulted in interactions with others, prevented isolation through socialization, and stimulated their daily lives (Komatsu et al., 2017).

## Discussion

This review, conducted in accordance with PRISMA guidelines, was designed to establish whether the social relationships fostered by some activities (physical, leisure, cognitive) can be a vector of better physical, mental and social quality of life and a better quality of life for the older people. We begin by discussing our results on the effects of these social relationships on the physical, cognitive, social and psychological dimensions of quality of life, as well as quality of life in general. We then discuss the difficulty of grasping the dimensions of the social relationships fostered by group activities and understanding exactly how they are related to the different dimensions of quality of life. Finally, we discuss the importance of grasping these dimensions for future research and the challenges we have identified as a result of this review.

First, the present review highlighted the benefits of practicing various group activities in terms of improving or preserving older people’s quality of life. Most of the studies included in this review reported improvements in the physical, cognitive, social and psychological dimensions of quality of life. At the physical level, there was an improvement in physical condition (flexibility, coordination, endurance, etc.; Yuen et al., 2011; Chang et al., 2014; Kim et al., 2015; Levinger et al., 2020), as well as an increase in practice time (Levinger et al., 2020), bringing an improvement in functional autonomy. At the cognitive level, there were improvements in executive attention, processing speed, global cognition, verbal fluency, and memory (Kamegaya et al., 2014; MacAulay et al., 2019). These results are in line with the literature, which shows that the practice of physical and/or cognitive activity is beneficial for the cognitive health of Colcombe et al. (2003); Gheysen et al. (2018) and Guo et al. (2020). Our review also showed that physical activity can benefit the social and psychological dimensions of quality of life, reporting the development of social support, the reinforcement of autonomy, social interactions, and an increase in perceived quality of life (Nadasen, 2008; Yuen et al., 2011; MacAulay et al., 2019), as well as the development of a sense of belonging, and the strengthening of self-esteem and confidence (McAuley et al., 2000; Yuen et al., 2011; Levinger et al., 2020). The studies in this review all confirmed the beneficial effects of practicing diverse activities on the different dimensions of quality of life, as well as on general quality of life. Several studies investigating the effects on quality of life in general showed that group activities have beneficial effects on physical, mental and social health, and contribute to the maintenance of functional health in Camic et al. (2014); Kanamori et al. (2016) and Gagliardi et al. (2019). However, some of the studies included in this review failed to find any beneficial effect of group activity in some domains, simply observing the maintenance of basic physical (Emery and Gatz, 1990; Kamegaya et al., 2014), cognitive (Emery and Gatz, 1990; Kamegaya et al., 2014; Pentikäinen et al., 2021) and social (Aday et al., 2006) abilities. Failure to find significant relationships with certain dimensions or subdimensions of quality of life can be explained by the fact that some of the studies included in this review had sample sizes that were too small to allow relationships between activity and health to be identified in older people. Moreover, we included both interventional and observational studies, which did not all use the same assessment procedures (pre- and postintervention assessments, assessment of the effect of an intervention program, group comparisons, comparisons of solitary versus group mode of practice, quantitative and/or qualitative assessments), potentially leading to a methodological bias, with the resulting diversity and heterogeneity of results.

Although the studies included in this review yielded important and interesting results, and tended to agree with the rest of the literature on the beneficial effects of practicing different types of activity on older people’s cognitive functions and quality of life (Kelly et al., 2017; Marsillas et al., 2017; Guo et al., 2020), they had several limitations. In particular, there was difficulty grasping the dimensions of the social relationships fostered by group activities. The studies included in this review highlighted the beneficial effects of practicing group activities on the different physical, cognitive, social, and psychological dimensions of quality of life, but the actual role of the social relationships and the ways in which they influenced quality of life were not explained. As their dimensions were only very weakly defined in these studies, if at all, these relationships were treated as contextual data, rather than processes. We are therefore not in a position to say how and under what conditions the social relationships fostered by group activities affect older people’s quality of life and describe its direct impact on the specific dimensions of quality of life. The same observation was made by Haslam, who argued that the contribution of social factors to health remains underestimated and underemphasized in the literature (Haslam et al., 2018). The present review did not enable us to shed any further light on the contribution of social factors to the physical, cognitive and psychological components of quality of life.

Wakefield et al. (2019) recently stressed that improving quality of life involves addressing the complex interaction between people’s health and their social worlds. According to the notion of *social cure*, membership of a social group can improve quality of life, but only if members identify with the group (subjective sense of belonging to the group in question; Haslam et al., 2014, 2018). This social cure notion suggests that social identities can provide psychological resources, including a sense of connection among group members that engenders a sense of trust, meaning and purpose in life, as well as social support from other group members to help cope with life’s stresses and strains (Haslam et al., 2005; Greenaway et al., 2015; Wakefield et al., 2019). It is therefore important to explore this notion in depth, in order to define the impact of social factors and their relationships with the physical, cognitive, social, and psychological dimensions of quality of life, in order to act effectively on the quality of life of older people and promote healthy aging.

Future research must demonstrate the importance of social factors for health and identify the precise relationships and interactions between social relationships and quality of life dimensions. We see at least two main challenges in this area.

### Challenge 1: relationship between practice environment, cognitive abilities, and perceived benefits of practice

We suspect that the positive effects of combined activities (simultaneously practicing physical and cognitive activities) on cognitive and general quality of life are influenced by the environment in which these activities are performed. We suspect that group practice and practice environments fostering social relationships and strong social interactions have positive benefits for older people’s general and cognitive health. This concerns the notion of environmental enrichment, namely, the stimulation of the brain by the physical, cognitive and social environment. We would expect older people to perform better on the various physical, cognitive and psychosocial assessments in conditions of group practice, as well as in an enriched environment favoring social relationships.

This hypothesis could be tested with a population of older people in three different practice environments: (1) combined (physical and cognitive) activity program at home; (2) combined activity program in a traditional group environment (gymnasium); and (3) combined group activity program in an enriched environment promoting social relationships and social interactions. The programs and the assessments would have to be identical across these three conditions, in order to test this hypothesis.

### Challenge 2: psychosocial factors as mediators of the relationship

A second challenge would be to identify some of the key psychosocial factors that make older people’s cognitive quality of life more or less sensitive to the effects of combined activity practice, as a function of the practice environment. We suspect that it is the fluctuation of social factors (e.g., self-esteem, perceived social support) involved during practice that mediate the relationship between practice environment and older people’s cognitive abilities and their perceived quality of life. The feeling of belonging to a group and the relationships developed during group activities may enhance social factors such as perceived self-esteem or perceived social support, which then positively mediate the relationship between practice environment and participants’ cognitive abilities and perceived quality of life.

These measures of psychosocial factors could be used as potential mediators in statistical analyses to test whether older adults who engage in combined activity in a habitual group environment and in an enriched environment where social relationships are fostered benefit more in terms of cognitive ability and are more likely to have better perceived quality of life.

## Conclusion

Although the present review confirmed the benefits of physical and/or cognitive group activity for older people, in general quality of life, the contribution of social factors to this relationship remains unknown. The contribution of this review is, however, to have highlighted the complexity of objectifying the contribution of these social relationships and the mechanisms that come into play during the practice of these activities and their benefit on quality of life. Further studies assessing the relationships and interactions between these social factors and cognitive ability and quality of life could help to expand on these findings and identify new ways of promoting healthy aging.

## Data availability statement

The original contributions presented in the study are included in the article/Supplementary material, further inquiries can be directed to the corresponding author.

## Author contributions

This study was initially drafted by TG. Subsequent version was reviewed by DC, GB, and CE. Revision were made by TG. All authors contributed to the article and approved the submitted version.

## Funding

This work was supported by Région Nouvelle-Aquitaine grant number [AAPR2021-2020-12021410].

## Conflict of interest

The authors declare that the research was conducted in the absence of any commercial or financial relationships that could be construed as a potential conflict of interest.

## Publisher’s note

All claims expressed in this article are solely those of the authors and do not necessarily represent those of their affiliated organizations, or those of the publisher, the editors and the reviewers. Any product that may be evaluated in this article, or claim that may be made by its manufacturer, is not guaranteed or endorsed by the publisher.
